# Nonalcoholic steatohepatitis: A comprehensive updated review of risk factors, symptoms, and treatment

**DOI:** 10.1016/j.heliyon.2024.e28468

**Published:** 2024-03-25

**Authors:** Feryal Savari, Seyed Ali Mard

**Affiliations:** a Department of Medical Basic Sciences, Shoushtar Faculty of Medical Sciences, Shoushtar, Iran; b Clinical Sciences Research Institute, Alimentary Tract Research Center, Department of Physiology, The School of Medicine, Ahvaz Jundishapur University of Medical Sciences, Ahvaz, Iran

**Keywords:** NASH, NAFLD, MASLD, Diagnosis, Management, Treatment

## Abstract

Non-alcoholic steatohepatitis (NASH) is a subtype of nonalcoholic fatty liver disease and a progressive and chronic liver disorder with a significant risk for the development of liver-related morbidity and mortality. The complex and multifaceted pathophysiology of NASH makes its management challenging. Early identification of symptoms and management of patients through lifestyle modification is essential to prevent the development of advanced liver disease. Despite the increasing prevalence of NASH, there is no FDA-approved treatment for this disease. Currently, medications targeting metabolic disease risk factors and some antifibrotic medications are used for NASH patients but are not sufficiently effective. The beneficial effects of different drugs and phytochemicals represent new avenues for the development of safer and more effective treatments for NASH. In this review, different risk factors, clinical symptoms, diagnostic methods of NASH, and current treatment strategies for the management of patients with NASH are reviewed.

## Abbreviations

CAP: Controlled attenuation parameter
LSM: liver stiffness measurement
CK18: Cytokeratin 18
CMEC: China Multi-Ethnic Cohort
FLIP: Fatty Liver Inhibition of Progression
HbA1c: Hemoglobin A1c
HCC: Hepatocellularcarcinoma
IR: Insulin Rresistance
MRE: Magnetic resonance elastography
NAFLD: Non-alcoholic fatty liver disease
NASH: Non-alcoholic steatohepatitis
MAFLD: Metabolic dysfunction-associated fatty liver disease
MASLD: Metabolic dysfunction-associated steatotic liver disease
PDFF: Proton density fat fraction
PNPLA3: patatin-like phospholipase domain containing the 3
TZDs: Thiazolidinediones
UDCA: Ursodeoxycholic acid

## 1 Introduction

Non-alcoholic fatty liver disease (NAFLD) involves a clinicopathological spectrum of liver diseases ranging from steatosis, non-alcoholic steatohepatitis (NASH), and fibrosis to cirrhosis [1]. Inflammatory and progressive NAFLD can induce NASH, which is characterized by the hepatic steatosis, hepatocyte ballooning, and lobular inflammation. According to studies, NASH patients are more likely than the general population to develop into hepatocellular carcinoma (HCC) [2]. Besides, NASH patients have an annual mortality 1.7 times higher than that of NAFLD patients [3]. NASH is one of the most common forms of chronic liver disease in developed countries, leading to a significant burden on healthcare systems worldwide. The global burden of NAFLD, and NASH in 2018 was approximately 25% and 3–5%, respectively [4]. Based on a recent meta-analysis, the prevalence of NAFLD has increased by 50%, from 25.3% in 2006 to 38.2% in 2019. The estimated global prevalence of NAFLD is approximately 30.1%, which is consistent with the increasing rates of obesity and type 2 diabetes [5].

The secret to stopping the advancement of the illness is now early detection and care, as well as the identification of NASH risk factors that contribute to the disease's higher prevalence rate and severe effects. Besides, the optimal treatment for NASH would decrease metabolic comorbidities, the risk of cardiovascular events, and liver-related mortality [6].

This review provides a clinical overview of NASH, focusing on its risk factors, symptoms, diagnosis, and latest treatment methods.

### 1.1 Investigation of the risk factors for NASH

**Fig. 1:**
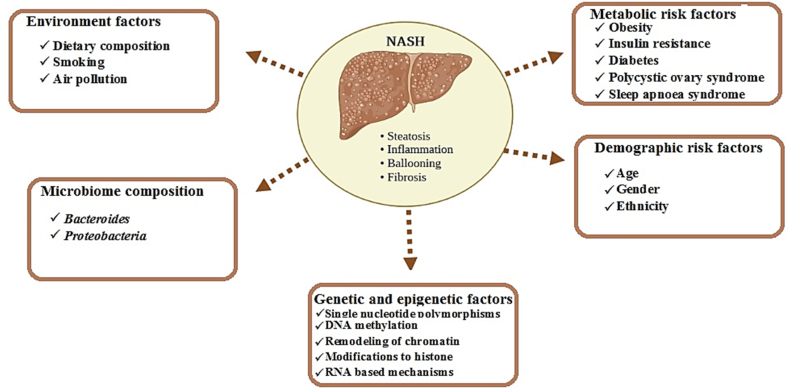
The risk factors that may induce non-alcoholic fatty liver disease (NAFLD) in the liver of patients.

In the pathogenesis of NAFLD and NASH, complex interactions among the metabolism and demography of patients, genetic variants, gut microbiota, and environmental factors can play a key role [7]. These factors and their complex interactions can be considered risk factors for the induction of NAFLD, and NASH (Fig. 1).

#### 1.1.1 Metabolic risk factors for NASH

A cluster of clinical features such as central obesity, dyslipidemia, hyperglycemia, and hypertension is called metabolic syndrome. Metabolic syndromes have been linked to a broad variety of metabolic abnormalities, such as low levels of anti-inflammatory adipokines, low-grade systemic inflammation with altered cytokines, and increased visceral adipose mass and free fatty acid levels [8]. The presence of metabolic syndrome in individuals with NAFLD, independent of age, gender, and body mass, can increase the risk of NASH threefold [9].

Obesity induced lipotoxicity and glucotoxicity can increase the risk of steatosis in the liver and its progression to NASH [10]. Previous studies showed that the prevalence of NASH in the individuals with normal weight was 2.7%, whereas it increased to 27–37% in morbidly obese subjects undergoing bariatric surgery [11]. It seems that the deposition of adipose tissue in the liver of obese patients can trigger an intra-hepatic inflammatory process [12]. Subsequently, innate immune cells in the liver are activated and release greater amounts of proinflammatory cytokines, such as tumor necrosis factor-α (TNF-α) and IL-6, which can lead to the onset of fibrogenic process [13]. Visceral adipose tissue in the liver is metabolically active and secretes adipokines (e.g., leptin and adiponectin) and hormones, which can contribute to the progression of NAFLD to NASH, cirrhosis, and HCC [13]. Therefore, the presence of visceral adipose tissue can be an indicator of hepatic inflammation, fibrosis, and advanced NASH [14].

One of the main pathogenic factors for the development of NASH and hepatocellular damage is insulin resistance (IR) [15]. Insulin normally inhibits lipolysis in adipose tissue, which lowers the content of VLDL or suppresses its synthesis in the liver [16]. In the subjects with NASH, the production of triglyceride-rich VLDL particles in the liver or lipolysis is not suppressed by insulin, leading to an increase in serum triglyceride levels [17]. Dyslipidemia is characterized by a reduction in HDL cholesterol and the production of small, dense LDL particles that are highly atherogenic [18]. Therefore, the patients have a higher risk of developing cardiovascular diseases. Insulin resistance in the subjects with NASH is higher than that in subjects with simple steatosis, and predicts fibrosis progression [19]. Studies show that liver histology is less severe in those with non-alcoholic fatty liver disease (NAFLD) who do not have IR. However, in this patient group, obesity was independently linked to substantial fibrosis irrespective of IR [20].

There is a strong relationship between diabetes and the risk of NASH [21,22]. Clinical cohorts have shown that impaired glucose tolerance can increase the risk of NASH by threefold [23]. Based on the autopsy studies, a 12.2% prevalence of NASH was reported in patients with diabetes, whereas it was only 4.7% in non-diabetic individuals [24]. An increase in the severity of diabetes parallels an increase in the risk of developing HCC and death from cirrhosis [25].

Polycystic ovary syndrome (PCOS) is a metabolic syndrome that is a risk factor for NASH severity and advanced fibrosis [26]. Disturbances in metabolism and high testosterone levels in women with PCOS may further promote NAFLD/NASH [26].

An increasing body of experimental data links NAFLD/NASH to obstructive sleep apnea syndrome (OSAS). Hyperglycemia and insulin resistance are typical sleep disorders that coexist with OSAS. Hepatic lipid peroxidation and NASH may be brought on by OSAS [27].

#### 1.1.2 Risk factors related to the demography of NASH patients

Some studies confirmed a strong correlation between increasing age and the prevalence of NASH as well as the development of NASH-induced fibrosis and HCC [28]. However, the duration of disease (and not age alone) is an important factor in the relationship between age and NASH development and/or severity that should be considered [29]. However, epidemiological studies reported that NASH and fibrosis are associated with increasing age, particularly after the fifth decade of life [10]. It is pertinent to note that more aggressive and rapid NAFLD progression was described in children than in adults [30].

It is yet unknown how gender affects the frequency of NASH. While some research suggests that NASH is more common in women than in men, other studies find the contrary [31]. Therefore, studies on larger populations in the future may help to understand the relationship between gender and NASH induction.

Multiple studies showed that the prevalence of NASH varies with ethnicity. Hispanics had the greatest frequency of NASH, which was notably greater than that of Caucasians. It seems that lifestyle and genetic predisposition are significant influences, however the exact cause for this discovery remains unclear [32].

#### 1.1.3 Risk factors of NASH related to genetic predisposition and epigenetic factors

Currently, there is strong evidence of the heritability of NASH and genetic and epigenetic factors associated with the progression and pathogenesis of the disease [33]. The major common genetic determinant of NASH is I148 M PNPLA3 variant, which is a missense mutation in the patatin-like phospholipase domain-containing 3 (PNPLA3) gene [34]. This gene can have a strong effect on the accumulation of hepatic fat and histopathological severity of hepatic injury. Single nucleotide polymorphisms in glucokinase regulator, neurocan, Transmembrane 6 Superfamily Member 2, Membrane-Bound O-Acetyltransferase Domain Containing 7, 17-Beta Hydroxysteroid Dehydrogenase 13, and lysophospholipase-like 1 genes are the other genetic variants related to NASH [35]. The cellular metabolism of lipids in the liver, elevated hepatic fat storage, and hepatic glucose absorption are all significantly impacted by these gene polymorphisms [36].

There is growing evidence that portrayed epigenetics (contribution of environmental and genetic factors) play an important role in the pathogenesis and progression of NASH [34,37]. Epigenetic modifications that occur by the alterations in DNA methylation, chromatin remodeling, histone proteins modifications, and RNA-based mechanisms, such as non-coding RNAs, can explain the effect of environmental factors on phenotypes. Thus, whereas common genetic variations cannot account for the missing heritability of NASH, epigenetics may partly explain it [34]. Embryo development can be affected by maternal obesity and diabetes, parental environment, dietary habits, lifestyle, and behavior that lead to extensive alteration of epigenome and chromatin structure of the offspring [34]. These factors are correlated with NASH development [34].

#### 1.1.4 Risk factors related to gut microbiome composition of NASH patients

Recent studies shown that gut microbiota can directly affect NAFLD and its progression to NASH. Through its impact on hepatic metabolism of fats and carbohydrates, the gut microbiota balances the pro- and anti-inflammatory actions of the liver [38]. NASH patients often exhibit obesity and bad eating habits. Therefore, the effects of diet and accompanying metabolic changes in the patients with NASH are difficult to distinguish from the effects related to the altered microbiome under the same conditions [39]. For example, the abundance of *Bacteroides* and *Proteobacteria* in the gut of NASH patients was reported to be higher than that in the healthy individuals [40]. Stool specimens were subjected to 16S rRNA gene microarray analysis in the children with NAFLD and showed more abundant Gammaproteobacteria (phylum *Proteobacteria*) and Prevotella (*Bacteroides*) than the microbiota of obese health children [41]. According to some research, treating NASH patients with the antibiotic rifaximin reduced the development of bacteria in the small intestine, which improved liver function [42]. Although these studies showed that there may be correlations between intestinal bacterial composition and NAFLD or NASH, the underlying mechanisms are not yet known.

#### 1.1.5 Risk factors of NASH related to the environment

Recently, the potential effects of some environmental factors on the development of liver diseases, such as NASH received increasing attention. Previous studies showed that dietary composition and lifestyle, especially in developed societies, may predispose individuals to NAFLD/NASH [43].

Numerous studies suggested that dietary composition plays an important role in the development of NAFLD/NASH. Consuming large quantities of sugar and fat in the diet increases the likelihood of developing steatosis and disrupts liver function via portal circulation [43]. Besides, NAFLD/NASH patients were more likely to have the least healthy eating habits, such as consumption of high-sodium and lower nutrients and fresh fruits [44]. In developed societies, the rise in knowledge-based jobs, combined with a decrease in the population's physical activity, leads to obesity and an increase in liver diseases, such as NASH [45].

The relationship between smoking and the incidence and progression of NASH was reported in several recent clinical studies [46]. It seems that smoking may exacerbate liver fibrosis in NASH patients via a variety of intricate routes, including oxidative stress and oncogenic signaling [46].

Ambient air pollution (AP), and the increased risk of liver disease are scientific topics that should be considered. While there is no epidemiological evidence linking air pollution to NAFLD/NASH, co-exposure to metals and phthalates—both of which are part of particulate matter—has been shown to disrupt liver biochemical pathways using multi-omics research [47]. Guo et al. (2022) reported a significant association between long-term exposure to ambient air pollution and NAFLD risk based on the China Multi-Ethnic Cohort (CMEC). Regarding the limited clinical data, the relationship between air pollution and NAFLD/NASH induction requires more detailed studies.

### 1.2 Clinical symptoms and diagnosis of NASH

The identification of pre-symptomatic patients at risk of NAFLD/NASH would be the best way to enable earlier disease intervention. Most patients with NAFLD/NASH are asymptomatic and usually have nonspecific symptoms. Frequent complaints of these patients included fatigue, anorexia, nausea, vomiting, malaise, headache, and vague right upper quadrant abdominal discomfort [48]. Hepatomegaly, splenomegaly, spider angiomata, raised serum triglyceride, impaired fasting glucose, high serum aminotransferase levels, moderate jaundice, and thrombocytopenia were among the physical examination findings of NASH patients [49]. Currently, invasive and non-invasive methods are used to more accurately diagnose and complement the overall appearance of NAFLD/NASH (Table 1).

**Table 1:** Clinical symptoms and diagnosis of NASH.

Clinical symptoms and diagnosis	Diagnostic criteria and evaluated factors
Liver Biopsy	Histopathological components and related scoring system NAS score [50] − Steatosis − hepatocellular injury − inflammation − fibrosis (the most relevant factor associated with NASH-related mortality) * The clinical utility of NAS score is questionable SAF score [50,51] − steatosis − disease activity (hepatocellular ballooning and lobular inflammation) − fibrosis * In clinical trials, the SAF score has not been tested.
Biomarkers	NASH Test (NT) parameters [52] − Height − Weight − age − gender − serum lipid profile − transaminases − α_2_-macroglobulin − haptoglobin − total bilirubin − apolipoprotein A1 − GGT NASH markers − CK18 (apoptosis marker) [53] − FGF21 (inflammation marker) [54] − TNF-α (non-specific inflammation marker) [55] − IL-6 (non-specific inflammation marker) [55] − IL-8 (non-specific inflammation marker) [55] − CRP (non-specific inflammation marker) [55] − FOXA1 (steatosis marker) [56] oxNASH score [57] − age − BMI − AST level
Imaging techniques (detection priority)	Ultrasonography (lesion microcirculation) [58,59] − Parenchymal alterations − Fatty infiltration − Dilatation of hepatic veins − Ascites FibroScan (Simple steatosis) [60] − hepatocyte steatosis − CAP score – steatosis − LSM-fibrosis MRI-PDFF (Liver fat content) [61] − Steatosis MRE (Liver stiffness) [62] − Steatosis − necroinflammation − Fibrosis − Cirrhosis

SAF: steatosis, activity, fibrosis; MRI: magnetic resonance imaging; MRI-PDFF: MRI proton density fat fraction; CAP: controlled attenuation parameter; LSM: liver stiffness measurement; FOXA1: transcription factor forkhead box protein A; FGF21: Fibroblast growth factor 21; GGT: gamma glutamyl transferase; CRP: C-reactive protein; TNF-α: tumor necrosis factor-α; IL: interleukin; NAS: NAFLD (Non-Alcoholic Fatty Liver Disease) Activity Score; NASH: Non-alcoholic Steatohepatitis; CK18: cytokeratin 18; MRE: Magnetic resonance elastography.

#### 1.2.1 Liver biopsy

Accurate evaluation of different possible histological components in a patient with clinical signs of NAFLD and the determination of their relationship can be achieved by conducting a liver biopsy [50]. Biopsy can provide more essential information than the presence or absence of NASH via a histopathological spectrum that includes a mixture of various lesions [50]. Four primary categories comprise the histological spectrum of non-alcoholic steatosis (NASH): steatosis, hepatocellular damage, inflammation, and fibrosis [50]. The liver biopsy method can help determine the relative contribution of comorbidities in the patients with NASH and assess the effect of drugs in controlled clinical trials [63]. Liver biopsy is a reliable method for the semiquantitative assessment of injury severity. However, it falls short in reflecting the histological complexity of chronic NASH owing to the continuous spectrum of histological lesions it displays [50]. Furthermore, the procedure is associated with potential complications that pose risks to patients.

NASH is a complex, multifactorial disease; thus, no single diagnosis method is likely to be omitted to predict clinical outcomes or benefits of therapy. Furthermore, the most critical stage in the development of severe liver disease, with a greater risk of fibrosis and a bad prognosis, is the switch from simple steatosis to NASH. Therefore, identifying the possibility of hallmark dynamic changes from NAFLD to NASH is an ongoing challenge. Bedossa et al. (2014) developed the Fatty Liver Inhibition of Progression (FLIP) algorithm, in which histological steatosis, disease activity, and fibrosis scores are useful [51].

#### 1.2.2 Fibrosis-4 index (FIB-4) and NAFLD fibrosis score (NFS)

Several surrogate markers, based on routine clinical and laboratory parameters, were developed to identify fibrosis in the patients with chronic liver disease. Notably, the fibrosis-4 index (FIB-4) and NAFLD fibrosis score (NFS) were extensively employed to predict liver fibrosis among large cohorts of patients with NAFLD [64,65]. However, despite their affordability and availability, the precise clinical utility of FIB-4 and NFS remains unclear even in resource-limited environments [66]. NAFLD is often linked to slightly increased hepatic transaminases in clinical practice. It is noteworthy, therefore, that there was little evidence of a relationship between high blood transaminase levels and liver histology [67,68]. Moreover, FIB-4 and NFS included serum transaminases in their calculations, however, it remains uncertain whether their diagnostic accuracy varies based on serum transaminase levels [68]. To further enhance diagnostic accuracy, recent studies suggested performing Fibroscan in NAFLD patients with advanced fibrosis following initial triaging with FIB-4 and NFS [69]. The FIB-4 score calculation serves as a non-invasive tool (NIT) for risk stratification of individuals at low risk of progressive liver disease. This allows primary healthcare providers to effectively manage cardiometabolic comorbidities in such individuals [70]. Combining Enhanced Liver Fibrosis (ELF) scores with FIB-4 may help identify patients who are appropriate for specialist care or clinical trials, as well as help weed out patients who have severe fibrosis, according to emerging research. However, additional studies are necessary to validate the utility of this combination and its role in clinical practice [71]. Furthermore, a previous recommendation was made regarding the combined utilization of NFS and FIB-4 scores alongside liver stiffness measurement (LSM). This combination shown enhanced diagnostic accuracy and reduced misclassification rates, particularly in the indeterminate zone of advanced fibrosis [72].

#### 1.2.3 Biomarkers

Although liver biopsy is still the standard procedure for diagnosing NASH, reliable diagnostic criteria that can be measured with less or even noninvasive techniques are urgently needed. Various molecular, cellular, hormonal, and blood biomarkers have been studied and created to reflect and identify the severity of nonalcoholic steatohepatitis (NASH) and its underlying pathways, in addition to histological assessment. The combination of several biomarkers, based on the complexity of NASH mechanisms, can provide an accurate diagnosis. Panels such as the Nash Test (NT) in NASH patients, including baseline patient characteristics such as height, weight, age, gender, and serum levels of cholesterol, triglycerides, transaminases, α_2_-macroglobulin, haptoglobin, total bilirubin, apolipoprotein A1, and GGT was used [52]. A novel machine learning approach uses age, HbA1c, gamma glutamyl transferase (GGT), adiponectin, apoptosis marker M30 (caspase-cleaved cytokeratin) and CHeK score to detect NASH and monitor its development from NAFLD to NASH [73].

When compared to NAFLD patients, the rise in cytokeratin 18 (CK18), an apoptosis marker, in the blood may indicate hepatocyte death by necroptosis and apoptosis [53]. Although CK18 is one of the most promising NASH biomarkers, its sensitivity and specificity for predicting NASH are 66% and 82%, respectively [74].

The level of fibroblast growth factor 21 (FGF21) in the blood is another marker. Decreased FGF21 levels are correlated with increased liver inflammation in patients with NASH [54].

Increased serum levels of TNF-α, several interleukins such as IL-6 and IL-8, and C-reactive protein (CRP) were proposed as clinical markers of NASH. However, they are not considered diagnostic markers of NASH because they are insensitive to NASH-specific inflammatory changes [55].

Forkhead box protein A (FOXA1), a transcription factor, is one of the most recent and sensitive non-invasive indicators of hepatic fat accumulation in NASH participants [56]. By reducing fatty acid absorption and IR, FOXA1 functions as an anti-steatotic drug in individuals with NAFLD/NASH [56]. FOXA1 is not secreted into serum, which limits using the FOXA1 as a diagnostic biomarker.

Oxidative stress, indicated by excessive ROS production, is one of the most critical mechanisms underlying NASH pathogenesis [75]. Calculation of lipid catabolism and *de novo* lipogenesis together with NASH patient characteristics, including age, BMI, and AST level, made a diagnostic biomarker called the oxNASH score [57]. Elevation of this biomarker may not be solely linked to circumstances generated by NASH/NAFLD, even if it does reflect alterations in liver lipid oxidation. Moreover, IR and metabolic syndrome may raise oxNASH readings [55]. In general, the best choice of specific serum markers for NASH is not optimal. Therefore, future studies are needed to better understand the underlying mechanisms and key molecules involved in the development and progression of NAFLD to NASH.

#### 1.2.4 Imaging techniques

Using imaging techniques in NASH diagnosis can provide direct information about the health status of the liver, and has therefore appeared to be an attractive alternative for assessing steatohepatitis.

Ultrasonography was one of the first imaging diagnostic tools for steatohepatitis. Parenchymal alterations, fatty infiltration, and dilatation of the hepatic veins and ascites were evaluated using ultrasonography [58]. Ultrasonography is a significant diagnostic add-on for screening patients at risk for NAFLD, particularly when liver enzymes are elevated. This technique supports precise diagnostic techniques for the identification of localized and diffuse liver diseases by providing qualitative monitoring and quantitative analysis of lesion microcirculation in the liver [59]. Ultrasonography showed no side effects. However, the sensitivity of ultrasonography is limited when hepatic steatosis content is below a certain threshold [59]. Traditional ultrasonography uses semi-quantitative ordinal classifications for liver fat, such as mild, moderate, and severe, which exhibit limited inter-observer agreement [76]. In contrast, contemporary quantitative ultrasound techniques offer enhanced performance using quantitative data derived from acoustic parameters of liver tissue. The attenuation parameter, backscatter coefficient, and ultrasonic wave speed/wavelength represent the extensively the evaluated quantitative characteristics [77]. Moreover, the amalgamation of these parameters has the potential to improve diagnostic accuracy [78].

FibroScan device with controlled attenuation parameter (CAP) is another imaging method that allows the detection of hepatic steatosis in NASH patients with approximately 10% fatty hepatocyte degeneration without being influenced by liver fibrosis or cirrhosis [60]. While this technique may effectively discriminate between grades of 10% and 33% steatosis, CAP rises after a patient's meal and may result in patient misclassification if the operator disregards preanalytical guidelines [79].

The quantification of hepatic fat content with high spatial resolution in patients with NASH can be performed using magnetic resonance imaging (MRI). Advanced MRI techniques, such as MRI proton density fat fraction (MRI-PDFF), were used to detect hepatic steatosis and assess liver fat over the entire liver [61]. PDFF is considered the best-suited quantitative MR-based biomarker for tissue fat concentration because it is robust, practicable, reproducible, and has widespread clinical implementation [80].

Recently introduced MRI Aspartate Aminotransferase (MAST) scores have been developed for the identification of subgroups among the patients with NASH/NAFLD. Aspartate aminotransferase (AST), magnetic resonance elastography (MRE), and proton density fat fraction obtained from magnetic resonance imaging (MRI-PDFF) are all included in this scoring system [81]. Furthermore, the MAST score showed accuracy in identifying individuals with at-risk NASH, particularly those at high risk of disease progression. Moreover, it outperformed other noninvasive methods, including NFS, FIB-4, and FibroScan-AST [81,82].

Magnetic resonance elastography (MRE) is a method used to detect progression and treatment response in patients with chronic NASH [83]. MRE has a high accuracy to determine liver stiffness and diagnosing advanced fibrosis and cirrhosis. When it comes to the diagnosis of steatosis and necroinflammatory activity in NAFLD patients, MRE performs better than transient elastography [62].

### 1.3 Current treatment strategies for the management of NASH

Although the global burden of NAFLD/NASH and its prevalence are growing, there are still no FDA-approved medications that are especially prescribed for this liver disease. As NASH has a complex pathogenesis, an effective treatment should be able to target multiple steps of this disease [84]. Currently, NASH management is based primarily on lifestyle modifications and hepatoprotective drugs (Table 2). Several natural bioactive compounds with promising outcomes have been investigated in experimental NASH models and in clinical trials [85]. The side effects of therapeutic interventions and their significant effects in patients with chronic NASH can limit patient compliance [86].

**Table 2:** Current treatment strategies for the management of NASH.

Treatment strategies	Evaluation Criteria
Lifestyle modification	diet and physical activity low-fat hypocaloric diet (calorie intake restriction, with a focus on reducing fat intake) and walking [87] ≥5% Weight loss (30%) ↓ NAS (47%) Fibrosis regression (19%) NASH resolution (25%) * The highest rates of improvement in histologic features of NASH occurred in patients with weight losses ≥10%. − Mediterranean diet (high content of dietary fiber with highest ratio of MUFAs to SFAs) [88] Weight loss (++) ↓ TC/HDL ↓↓ Fasting Glc ↓ Insulin level ↓ Leptin ↓ HOMA-IR ↓ CRP − Low-carbohydrate diet (low content of carbohydrate and high contents of fat, protein and cholesterol) [88] Weight loss (+++) ↓ TC/HDL ↓ Leptin ↑ Fasting glucose ↓ CRP − Low-fat diet [88] Weight loss (+) ↓ TC/HDL HOMA-IR (no change) Insulin level (no change) ↓ Fasting Glc (no change) ↓ CRP ↓ Leptin
Bariatric surgery	− Gastric bypass (RYGB) − Gastric banding (AGB) * RYGB is more effective in weight loss and improvement of NAFLD parameters [89]: ↓ Fasting blood Glc (RYGB > AGB) ↓ HOMA-IR (RYGB > AGB) ↑HDL cholesterol (no SD) ↓ Triglycerides (RYGB > AGB) ↓ ALT (no SD) ↓ AST (AGB > RYGB) ↓ GGT (RYGB > AGB) ↓ ALP (no SD) ↓ TB (RYGB > AGB) ↓ NAS (RYGB > AGB) ↓ Steatosis (RYGB > AGB)
Pharmacological options	Insulin Sensitization F095 TZDs [90] ↑insulin sensitivity ↓ AST and ALT Histological improve Altering Lipid Metabolism F095 Ursodeoxycholic acid (UDCA) [91] No histological changes, LFT (no change) ↓ Aminotransferase and necroinflammation ↓ Apoptosis (MAPK pathway stimulation) ↓ FibroTest scores Serum hyaluronic acid (no change) ↓ Insulin Resistance ↓ Serum TNF-α Reducing Oxidative Stress F095 Antioxidants (vitamin E) [92] ↓ LFT (fibrosis score not change) Bilirubin (no change) Fasting serum Glcuse (no change) IR (no change) BMI (no change) Histological improve
Herbal medicine option	Curcumin [93] ↓ AST, ALT ↓ Steatosis ↓ BMI Lipid profile (serum) ↓ Fasting Glc ↓ Hb A1c Ultrasonographic findings: ↑hepatic vein flow ↓ portal vein diameter ↓ liver volume ↓ liver fat content Epigallocatechin-3-gallate (EGCG) [94] Weight loss ↓ BMI ↓ ALT ↓ AST ↓ ALP ↓ HOMA-IR ↓ TC, TG, LDL ↑HDL ↓ hs-CRP ↑Adiponectin Ginger [95] TAG ↓↓ LDL-C ↓ TG ↓ TC (no SD in some cases) ↑HDL-C (no SD in some cases) ↓ MDA ↑CAT ↑SOD ↓ ALT/AST ↓ Fasting blood Glc

LFT: liver function test; TZDs: Thiazolidinediones; IR: Insulin Resistance; MUFAs: monounsaturated fatty acids; SFAs: saturated fatty acids; SD: significant difference; ALP: alkaline phosphatase; ALT, alanine aminotransferase; AST: aspartate aminotransferase; BMI: body mass index; GGT:γ -glutamyltransferase; HDL, high-density lipoprotein; HOMA-IR, HOmeostasis Model Assessment of Insulin Resistance; TNF-α: Tumor Necrosis Factor-alpha; MAPK: mitogen-activated protein kinases; TB: total bilirubin; RYGB: Roux-en-Y gastric bypass; AGB: adjustable gastric banding; HbA1c: glycated hemoglobin; TC: total cholesterol; TG: triglycerides; LDL-Cholesterol: low density lipoprotein cholesterol; HDL-cholesterol: high density lipoprotein cholesterol; hs-CRP: high sensitivity C-reactive protein; TAG: triacylglycerol; SOD: superoxide dismutase; MDA: malondialdehyde; CAT: catalase; Glc: glucose.

#### 1.3.1 Lifestyle modification

The first step in NAFLD/NASH management is lifestyle modification (diet and physical activity), which is recommended in all guidelines [96]. It has been reported that resolution of NASH is observed in 65–90% of patients achieving ≥7% weight loss [87]. Weight loss has demonstrated efficacy in promoting the resolution of hepatic steatosis and fibrosis, even in lean individuals with NAFLD [97]. Nonetheless, patient compliance and adherence to dietary interventions pose challenges [97]. A restricted calorie diet can improve insulin sensitivity and optimize endogenous glucose synthesis [98]. However, specific diets are recommended for NASH resolution. It seems that Mediterranean and low-carbohydrate diets can be effective alternatives to low-fat diets, with different metabolic effects in NASH patients [88]. The results of the pilot study revealed that the prescribed diet consisting of 30–40% fat (especially monounsaturated and polyunsaturated fatty acids) and 40–45% carbohydrates (especially complex carbohydrates with fiber) in 15 patients reduced the histological markers of NASH [99]. Another clinical study has recommended the Mediterranean diet as a safe and inexpensive therapeutic option for children with obesity and NAFLD/NASH [100].

Besides diet, physical activity is also part of lifestyle changes. Although some recent studies have reported that exercise can reduce steatosis and liver stiffness [101], the additive effect of exercise on hepatic steatosis has not been well established. The investigation of the effect of physical activity on the resolution of NASH must be based on realistic and sustainable objectives [101]. Obtaining long-term compliance, particularly in patients who are not accustomed to regular intense exercise, is the main challenge.

#### 1.3.2 Bariatric surgery

Bariatric surgery can be performed to assist in weight loss and ameliorate metabolic complications in obese patients when lifestyle modifications and pharmacological treatment are not effective [102]. The National Institutes of Health recommends bariatric surgery for motivated candidates for severely obese patients (body mass index ≥40 or 35–40 with comorbidities). Previous studies have reported that NASH disappeared in patients (approximately 85–90%) who underwent bypass surgeries or gastric banding [103]. Evidence has showed that gastric bypass in a patient with NASH is more effective than gastric banding because gastric bypass can lead to greater weight loss in patients [89]. The effects of bariatric surgery in patients with NASH include a reduction in steatosis, mainly within the first year after surgery until 5 years later [104]. Although bariatric surgery can improve hepatosteatosis in patients for whom lifestyle therapy has failed to treat NASH, perioperative risks limit its application. Bariatric surgery is not recommended for patients with established cirrhosis because they have a higher risk of perioperative mortality [105]. However, the effects of bariatric surgery on fibrosis and necroinflammation in NASH patients are controversial. Therefore, prospective studies are required to answer these questions.

#### 1.3.3 Pharmacological options

Most current pharmacological treatments for NASH are prescribed for patients with fibrosis, cirrhosis, metabolic syndrome, and those aged >50 years. As no medication for the treatment of NASH has been approved, the risks and benefits of all prescribed medications should be weighed carefully.

Therefore, the genetic or pharmacological inhibition of apoptosis has been shown to reduce NASH progression. These initiatives could expedite drug development for the treatment of NASH, and subsequent cirrhosis and HCC prevention [96].

##### 1.3.3.1 Insulin sensitization

Insulin resistance plays an important role in NAFLD/NASH pathogenesis. Therefore, the use of insulin sensitizer drugs such as metformin and thiazolidinediones in patients with NASH may be useful [106]. Metformin is a biguanide agent that can decrease fasting levels of glucose, postprandial glucose, and glycosylated hemoglobin in obese patients with type 2 diabetes via reducing gluconeogenesis [107]. Several studies have reported that metformin has limited beneficial effects on the histopathological features of NASH [108]. Some studies have shown that metformin can improve liver function modestly and transiently decrease AST and ALT levels but has no histological benefit in NASH patients with steatosis, fibrosis, and inflammation [109].

Thiazolidinediones (TZDs: pioglitazone and rosiglitazone), which act as peroxisome proliferator-activated receptor gamma (PPAR-γ) agonists, are approved medications for type 2 diabetes, usually as second-line options [110]. Systematic reviews, histological and meta-analyses studies have revealed that TZDs can lower serum transaminases and improve insulin sensitivity, liver steatosis, lobular inflammation, and ballooning in NASH patients [90]. The use of pioglitazone in the treatment of NASH has been approved by several guidelines [96]. Despite the beneficial effects of TZDs, long-term treatment results in significant weight gain that may affect patient compliance [111].

##### 1.3.3.2 Altering lipid metabolism

Statins are considered a potential therapeutic option for NASH, which is commonly associated with dyslipidemia and characterized by elevated plasma and hepatic lipid levels [112]. The use of statins in NASH patients can protect them against cardiovascular events, which are the primary cause of mortality in NASH patients [113]. Based on many guidelines, prescribing statins to NAFLD patients with dyslipidemia and atherogenic profile is recommended [114]. Prescribing statins to NASH patients is safe, except for patients with decompensated cirrhosis and acute liver failure [115].

As dysregulation of pro-inflammatory factors and abnormal lipid metabolism contribute to the progression of NASH, exogenous administration of non-toxic bile acids such as ursodeoxycholic acid (UDCA) may be useful [91]. Previous studies have shown that the anti-apoptotic and anti-inflammatory effects of UDCA in the treatment of NASH can positively affect inflammation and IR [116].

Glucagon-like peptide 1 receptor agonists (GLP-1 RAs), also known as incretin mimetics, have many beneficial effects on obesity and inconsistent effects on hepatic enzymes in previous studies [117]. Although some studies have shown a short-term effect of liraglutide administration on decreasing fibrosis progression [118], several guidelines have demonstrated that the efficacy of these drugs against NAFLD/NASH is insufficient to recommend their use [96].

##### 1.3.3.3 Reducing oxidative stress

Oxidative stress increases cell death and fibrogenesis, which leads to NASH. Therefore, antioxidant compounds, such as vitamin E, may be promising treatment options for NASH. Previous studies have shown that vitamin E can decrease serum transaminase levels and improve histopathological features of NASH [92]. However, some guidelines do not recommend the use of vitamin E in patients with NASH owing to insufficient evidence regarding its efficacy [96].

Pentoxifylline is a xanthine derivative with radical-scavenging properties and is used to treat muscle pain in patients with peripheral artery disease [119]. Some studies have shown that pentoxifylline can induce a positive effect on steatosis and hepatocyte ballooning in patients with NASH [120].

#### 1.3.4 Herbal medicine option

Currently, the investigation and use of herbal medicines and their derivatives for the treatment of NASH has gained attention owing to their high safety, efficacy, and abundance [121]. Many herbs are known for their hepatoprotective properties and have been used as single agents or combination formulae in clinical trials [122].

##### 1.3.4.1 Silymarin

Silymarin is a bioactive compound obtained from the flowering tree, Milk thistle (*Silybum marianum*). Silymarin exhibits significant antioxidant and hepatoprotective effects [121]. Milk thistle extract contains several flavonolignans and polyphenols with hepatoprotective and antioxidant properties [123]. The results of a double-blind, placebo-controlled RCT of Silymarin (700 mg thrice daily for 48 weeks) in a Malaysian population with biopsy-proven NASH showed significantly decreased fibrosis and lowered serum ALT and AST levels compared to placebo. However, Silymarin has no significant effect on steatosis and inflammation in NAFLD patients [124]. The results of multi-center and phase II RCT showed that silymarin administration did not histologically improve non-cirrhotic NASH [125]. To date, no clinical studies have confirmed the effectiveness of silymarin as a primary bioactive compound for improving or retarding NASH progression. Therefore, futher studies are needed to determine the optimum dose and duration for the use of this compound in patients with NAFLD/NASH.

##### 1.3.4.2 Curcumin

Curcumin is a natural polyphenol found in the rhizome of Curcuma longa that has antioxidant, anti-inflammatory, and anti-fibrotic properties. The impact of curcumin on liver fibrosis in humans is not completely understood, but some clinical trials have demonstrated that administration of curcumin can decrease aminotransferase levels and ameliorate hepatic steatosis in NAFLD patients [93]. Although curcumin administration has shown good tolerance and safety in clinical trials, some individual case reports allude to the potential of curcumin in idiosyncratic liver injury [126]. Therefore, further scrutiny of curcumin safety in NASH patients is required. Owing to the absence of substantial clinical evidence supporting curcumin efficacy in these patients, the potential risk of the use of these compounds outweighs the hypothesized benefit.

##### 1.3.4.3 Resveratrol (RSV)

Resveratrol (RSV) is a natural polyphenol extracted from fruits, such as red grapes, blueberries, and blackberries, with well-known anti-inflammatory and antioxidant effects [127]. However, the clinical benefits of RSV in patients with NASH have been inconsistent. A clinical study reported that RSV in the treatment of NAFLD showed no improvement in parameters compared to placebo [128]. However, another clinical study reported contradictory results in patients [129]. Based on a recent meta-analysis, there is insufficient evidence regarding the use of RSV in the management of NAFLD/NASH [130]. Therefore, the use of RSV in the treatment of NAFLD/NASH requires conclusive evidence.

##### 1.3.4.4 Epigallocatechin-3-gallate (EGCG)

Epigallocatechin-3-gallate (EGCG) is a polyphenolic catechol derived from the leaves of the Chinese tea tree, *Camellia sinensis*. EGCG has antioxidant and anti-inflammatory effects on hepatocytes and hypolipemic effects [131]. Clinical studies have reported that green tea extract could significantly improve biochemical and radiologic markers of steatohepatitis compared with placebo [94].

##### 1.3.4.5 Ginger

Ginger is a commonly used food spice obtained from *Zingiber officinale*, and is used in traditional medicine for many diseases [95]. The beneficial effects of ginger on diabetes, obesity, and dyslipidemia have been confirmed in several studies [95]. Many clinical aspects of NAFLD/NASH, such as insulin-sensitive effects, stimulation of the antioxidant system, antidyslipidemic activities, and reduced hepatic fat content, can be induced by ginger administration [132]. However, the clinical application of ginger requires further clinical trials.

### 1.4 NAFLD or MAFLD

Metabolic dysregulation appears to be critically involved in the pathogenesis of NAFLD progression despite its nomenclature, focusing on the absence of alcohol abuse and excluding other secondary causes of steatosis [133].

Several naming and pathophysiologic drawbacks have been identified, which led an international panel of experts to propose renaming Nonalcoholic Fatty Liver Disease to Metabolic (dysfunction)-associated Fatty Liver Disease (MAFLD) to unravel many aspects of disease diagnosis and consider metabolic criteria, such as obesity, diabetes, and metabolic dysregulation [134]. Knowing the risk factors for concomitant diseases allows for timely screening and multilateral management of the disease spectrum [135].

Therefore, the term ‘non-alcoholic’ is not helpful in describing this disease. As an alternative to NAFLD, the term "MAFLD" better reflects the etiology of the disease, and there is wide consensus regarding this term. However, there are few reports in the prevalence of MAFLD and its renaming effect in improving diagnosis and primary care settings in the corresponding population [134].

Metabolic dysfunction-associated steatotic liver disease (MASLD) is an updated nomenclature endorsed by international experts for the condition previously known as NAFLD [136]. MASLD is used in patients with hepatic steatosis and exhibits at least one of five specific cardiometabolic risk factors [137]. Body mass index, blood pressure, plasma triglycerides, plasma HDL-cholesterol, and fasting serum glucose based on the presence of hepatic steatosis were defined as cardiometabolic risk factors within the MASLD framework [138].

These risk factors distinguish them from metabolic factors identified in the MAFLD framework. In cases where metabolic dysfunction is strongly suspected despite the absence of cardiometabolic risk factors, the term "possible MASLD" may be provisionally employed until further tests (e.g., oral glucose tolerance test and homeostasis model assessment of insulin resistance) are performed [137].

Therefore, guidelines are needed for precise assessment of MAFLD diagnostic criteria based on demographic, traditional, and metabolic risk factors, screening, and treatment [139].

It seems that preventing confusion, lack of clarity, and minimizing knowledge gaps among practitioners can help elucidate and define the frameworks and care models more precisely, as well as the management of the disease [135].

## 2 Conclusion

NASH development and progression are complex processes that involve multiple genetic, metabolic, and nutritional factors. In addition, the diagnosis of NASH is also a challenge as it requires confirmation using an invasive method. It seems that the treatment and management of NASH needs to target multiple metabolic abnormalities to be effective. Although approved medications specifically for the management of NASH are not yet available, several interventions have been implemented to ameliorate some aspects of NASH. Interventions, such as adopting a healthy lifestyle, medications, and surgeries with incomplete degrees of effectiveness, can be used. The use of herbal-based treatments is a promising and novel approach that is currently attempting to achieve a safe and effective treatment of NASH.

## Funding

No funding was received to assist with the preparation of this manuscript.

## Data availability

Not applicable.

Has data associated with your study been deposited into a publicly available repository?

No data was used for the research described in the article.

## CRediT authorship contribution statement

**Feryal Savari:** Writing – review & editing, Writing – original draft, Validation, Resources, Investigation, Formal analysis, Data curation. **Seyed Ali Mard:** Writing – review & editing, Conceptualization.

## Declaration of competing interest

The authors declare that they have no known competing financial interests or personal relationships that could have appeared to influence the work reported in this paper.
